# Pathophysiology of bradykinin and histamine mediated angioedema

**DOI:** 10.3389/falgy.2023.1263432

**Published:** 2023-10-18

**Authors:** Hermenio Lima, Jiayue Zheng, Dennis Wong, Susan Waserman, Gordon L. Sussman

**Affiliations:** ^1^ LEADER Research Inc., Hamilton, ON, Canada; ^2^ Department of Medicine, McMaster University, Hamilton, ON, Canada; ^3^ McMaster University, Hamilton, ON, Canada; ^4^ Division of Clinical Immunology and Allergy, Department of Medicine, University of Toronto, Toronto, ON, Canada; ^5^ Division of Clinical Immunology and Allergy, Department of Medicine, McMaster University, Hamilton, ON, Canada; ^6^ Department of Medicine and Division of Clinical Immunology & Allergy, University of Toronto, Toronto, ON, Canada

**Keywords:** angioedema, pathophysiology, complement and kinin pathways, vasoactive mediators, targeted therapies

## Abstract

Angioedema is characterized by swelling localized to the subcutaneous and submucosal tissues. This review provides an overview of angioedema, including the different types, triggers, and underlying pathophysiologic mechanisms. Hereditary and acquired angioedema are caused by dysregulation of the complement and kinin pathways. In contrast, drug-induced and allergic angioedema involve the activation of the immune system and release of vasoactive mediators. Recent advances in the understanding of the pathophysiology of angioedema have led to the development of targeted therapies, such as monoclonal antibodies, bradykinin receptor antagonists, and complement inhibitors, which promise to improve clinical outcomes in patients with this challenging condition. To accurately diagnose and manage angioedema, an understanding of this condition's complex and varied pathophysiology is both necessary and critical.

## 1. Introduction

Angioedema (AE), a condition characterized by sudden, self-limiting, localized swelling of the skin and mucosal tissues, presents a complex clinical challenge. Though isolated angioedema is possible, particularly in bradykinin-induced angioedema, angioedema is much more commonly associated with urticarial disorders such as chronic spontaneous urticaria (CSU) (1, 2).

AE is principally categorized into histamine-mediated angioedema and the rarer, but clinically significant, bradykinin-mediated angioedema. The pathophysiology of these types differs fundamentally and understanding these differences is essential to devising accurate diagnostic and therapeutic strategies (3).

Histamine-mediated angioedema is the more common variant of AE. It often manifests as an immediate type I hypersensitivity reaction, affecting the face, lips, tongue, and throat, and may be accompanied by urticaria or hives. Histamine-mediated angioedema typically responds well to standard allergy treatments such as antihistamines, corticosteroids, and epinephrine (4). In contrast, bradykinin-mediated angioedema can be precipitated by stress, trauma, or certain medications, often without any identifiable trigger (5).

Bradykinin-mediated angioedema is a condition marked by fluid extravasation due to vasodilation and increased vascular permeability, stimulated by bradykinin, a potent vasodilator. Its pathophysiology revolves around the complex interplay between bradykinin, high molecular weight kininogen (HMWK), and kallikrein (6). Disruption in this balance leads to characteristic symptoms such as nonpitting, nonpruritic, asymmetrical and localized swelling or the skin and/or mucosa; gastrointestinal mucosa involvement, for instance, may result in abdominal pain, nausea, vomiting, or diarrhea (7). Bradykinin-mediated angioedema's pathogenesis, pathophysiology, and clinical manifestations are still not fully comprehended, and mismanagement can lead to fatal outcomes (8).

This review focuses on a simple overview that aims to describe bradykinin- and histamine-mediated angioedema's pathophysiology.

## 2. Histamine-mediated angioedema pathophysiology

Histamine-mediated angioedema is a commonly encountered condition in emergency departments, accounting for nearly 40%–50% of angioedema cases. Though this reaction is mostly self-limited, laryngeal involvement in severe acute reactions such as anaphylaxis can be life-threatening due to the risk of asphyxiation (9).

The most well-characterized mechanism of histamine-mediated angioedema is a type I hypersensitivity reaction. During the sensitization phase of a type I hypersensitivity reaction, exposure to allergens (including food allergens such as milk or wheat) prompts an increase in the secretion of antigen-specific immunoglobulin E (IgE) molecules by plasma cells. During this asymptomatic reaction, IgE molecules bind to high-affinity Fc*ε*RI receptors which are constitutively expressed on mast cells and basophils (10, 11).

Re-exposure to the same allergen leads to IgEs cross-linking with the allergen, thereby triggering the degranulation of mast cells and basophils. This signals the “early-phase” of a type I hypersensitivity reaction (10). Inflammatory mediators such as biogenic amines (e.g., histamine) and serine proteases (e.g., tryptase and chymase) are released, and disrupted vascular integrity ensue via dilation and opening of endothelial cell junctions (10, 12). The resultant vasodilation and capillary permeability results in fluid accumulation in interstitial tissue spaces, causing non-pitting edema; predominantly, the face, ears, throat, tongue, lips, hands, feet, and genitalia are affected (10, 13).

The “late-phase”, on the other hand, is not necessarily dependent on IgEs. In contrast to early-phase reactions, cutaneous manifestations of a late-phase reaction involve accumulation and infiltration of eosinophils, neutrophils, CD4+ T cells, and basophils. Late-phase reactions occur more slowly than early-phase reactions, typically occurring hours rather than minutes after re-exposure to the antigen (14).

In addition to type I hypersensitivity reactions, histamine-mediated angioedema can also be caused by direct mast cell or basophil activation, resulting in the release of inflammatory mediators. Such direct activation can be caused by either endogenous or exogenous factors. Anaphylatoxins (complement fragments C3a, C4a, and C5a) are examples of such endogenous factors; they cause direct mast cell activation and degranulation by binding to non-Fc*ε*RI receptors on mast cells’ cell membrane. Iodine- and gadolinium-based contrast agents, on the other hand, are examples of exogenous factors which act directly on mast cells’ and basophils’ cell membranes to cause degranulation (15, 16).

Furthermore, disruption of the arachidonic acid pathway may cause histamine-mediated angioedema. The most notable example is NSAIDs-induced urticaria/angioedema (NIUA). Nonsteroidal anti-inflammatory drugs (NSAIDs) strongly inhibit cyclooxygenase-1 enzymes (COX-1), disrupting the arachidonic acid pathway. Such a disruption causes increased production of eosinophils, mast cells, and proinflammatory mediators; this, in turn, results in increased production of cysteinyl leukotrienes (a family of inflammatory lipid mediators) (17). Cysteinyl-leukotrienes increase vascular permeability, and in-vitro studies suggest that cysteinyl leukotrienes can induce histamine hyperresponsiveness by increasing the expression of histamine receptors (18, 19). Altogether, angioedema ensues.

## 3. Bradykinin-mediated angioedema pathophysiology

The pathogenesis of bradykinin-mediated angioedema was initially believed to be dependent on C1-inhibitor (C1-INH) deficiency and complement activation. A peptide called “C2-kinin” derived from C2 was proposed as the cause of angioedema (20). However, further research showed that this peptide could not be generated from purified components, and it was discovered that bradykinin was the only vasoactive peptide produced in the plasma of patients with hereditary angioedema (HAE) (21, 22). Elevated levels of bradykinin were found in HAE patients during angioedema attacks, confirming it as the mediator of swelling (22, 23).

**Figure 1:**
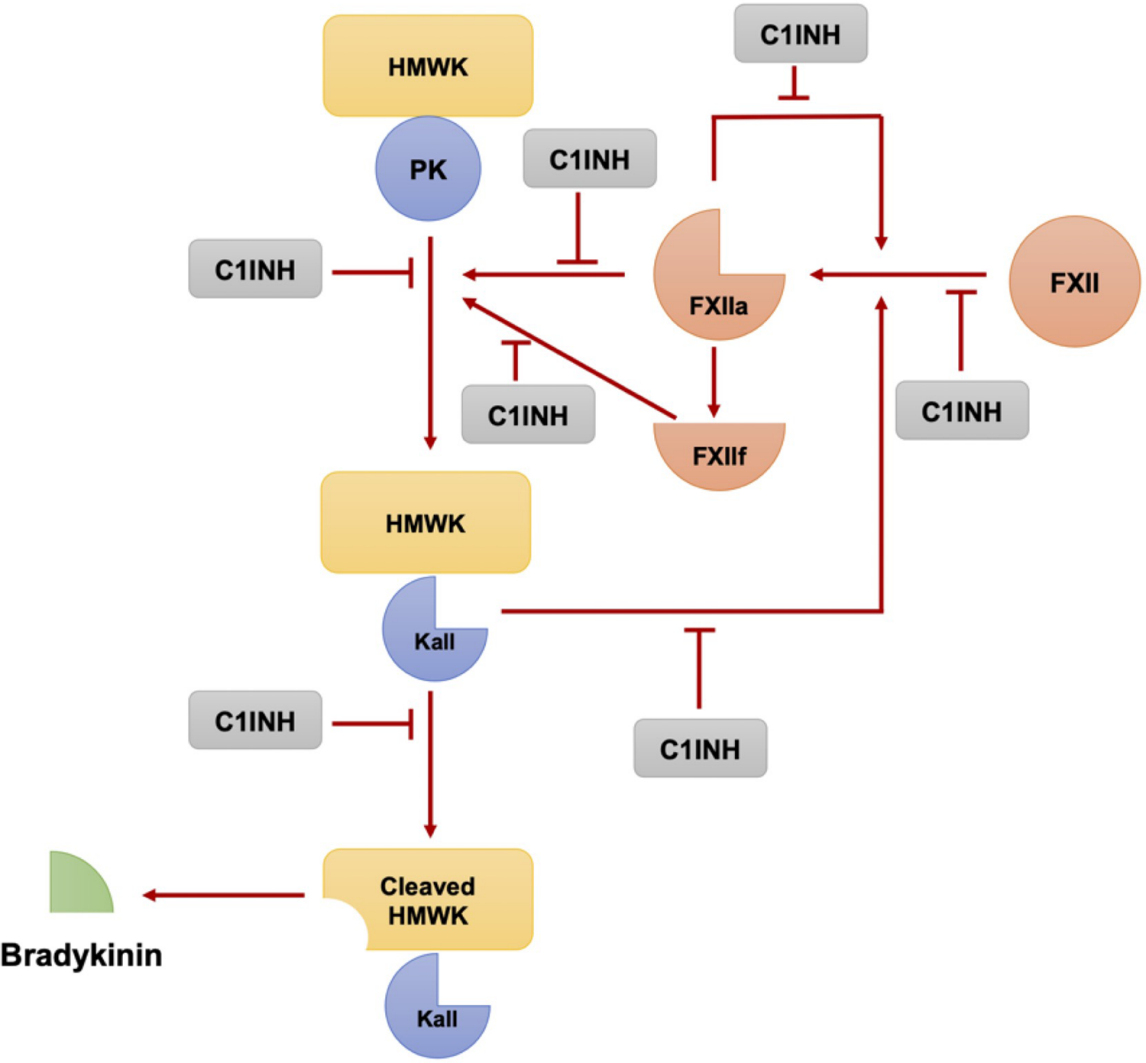
The plasma kallikrein-kinin and contact activation systems involve a series of reactions where Factor XII and plasma prekallikrein autoactivate to form factor XIIa and plasma kallikrein respectively. These, in turn, can further activate each other, amplifying the pathway. Moreover, XIIa creates XIIf, which can also convert prekallikrein to kallikrein. This cascade leads to the production of bradykinin from high molecular weight kininogen, a process regulated by the C1 esterase inhibitor. (Modified from Pathophysiology of Angioedema. Dennis Wong, Hermenio Lima, Susan Waserman, Gordon L. Sussman, 2023 in print).

The key enzymes involved in bradykinin formation are activated factor XII and plasma kallikrein, both of which are inhibited by C1-INH (24, 25). C1-INH has multiple functions relevant to bradykinin formation, including the inhibition of factor XIIa, plasma kallikrein, and coagulation factor XIa. It is also involved in the regulation of complement activation (26). In the absence of C1-INH, there is overproduction of bradykinin due to the uncontrolled activity of these enzymes (Figure 1) (25, 27).

Factor XII has a small amount of enzymatic activity, which is sufficient to initiate the bradykinin-forming cascade when it encounters negatively charged macromolecules (28). Once activated, factor XIIa can activate factor XI, which continues the intrinsic coagulation cascade, and can also convert plasma prekallikrein to kallikrein. Kallikrein then digests high molecular weight kininogen (HMWK) to release bradykinin (25, 28). There is a positive feedback loop in which plasma kallikrein rapidly converts factor XII to factor XIIa, amplifying the activation of the cascade (29). Additionally, there is a fibrinolytic pathway that involves the conversion of plasminogen to plasmin by kallikrein, factor XIa, and factor XIIa. This pathway is important in the context of bradykinin-mediated angioedema (specifically hereditary angioedema with normal C1-INH activity), as plasmin can cleave and activate factor XII, thereby feeding the bradykinin-forming cascade (30).

The binding of all the components of the bradykinin-forming cascade to endothelial cells suggests that the endothelium may play a role in the initiation of angioedema attacks (31). Factors such as heat shock protein 90 (HSP-90) and prolylcarboxypeptidase, released by endothelial cells, can activate the HMWK-prekallikrein complex and contribute to the generation of bradykinin (32, 33).

It is important to consider bradykinin-mediated angioedema as a differential diagnosis, and hereditary angioedema (HAE) and certain drug-induced angioedemas (such as ACE-inhibitor-induced angioedema) are possible causes to be considered (34, 35).

HAE is characterized by impaired C1-INH activity, either due to a deficiency (type I) or dysfunction (type II) of the C1-INH protein (36, 37). There are also forms of HAE with normal C1-INH activity, which can be associated with mutations in various genes, including factor XII (38, 39).

In addition to HAE, bradykinin-mediated angioedema can be caused by certain medications, such as angiotensin-converting enzyme inhibitors (ACE inhibitors) and dipeptidyl peptidase-4 inhibitors (gliptins) (40, 41). These drugs decrease the breakdown of bradykinin, leading to its accumulation and the development of angioedema (42, 43). Differentiating between these causes may require genetic testing and evaluation of family history (44).

In summary, the pathogenesis of C1-INH deficiency involves uncontrolled activation of the bradykinin-forming cascade, leading to elevated levels of bradykinin and angioedema (45). Factors such as factor XII, plasma kallikrein, and endothelial cell components play important roles in this process (7). Understanding the underlying mechanisms of bradykinin-mediated angioedema is crucial for accurate diagnosis and appropriate management of affected individuals (46).

## 4. Discussion

Angioedema, a condition causing localized swelling in subcutaneous and submucosal tissues, can be classified into histamine-mediated angioedema and the less common but clinically significant bradykinin-mediated angioedema. Histamine-mediated angioedema can be either IgE-dependent (type I hypersensitivity reaction) or IgE-independent (e.g., direct mast cell and basophil activation, disruption of the arachidonic acid pathway). Bradykinin-mediated angioedema, on the other hand, results from an imbalance in the interplay of bradykinin, HMWK, and kallikrein. Understanding the pathophysiology of these types of angioedema is critical for diagnosis and management of this condition.

The differentiation between bradykinin-mediated angioedema and histamine-mediated angioedema is challenging due to an overlap of symptoms. Nonetheless, certain differences in clinical characteristics may guide physicians towards the correct underlying pathophysiology. The presence of pruritus is an important differentiating factor: with histamine-mediated angioedema, the sensation of pruritus is caused by excitation of certain histamine-sensitive unmyelinated C-fibers by histamine (47). Bradykinin-mediated angioedema, on the other hand, is typically non-pruritic. In addition, while patients with bradykinin-mediated angioedema may present with abdominal symptoms such as vomiting and diarrhea, such presentations are rare with histamine-mediated angioedema. Furthermore, while patients with histamine-mediated angioedema respond to treatment by antihistamines, corticosteroids, or epinephrine, patients with bradykinin-mediated angioedema do not respond to such treatments (48).

Differentiating between bradykinin-mediated angioedema and histamine-mediated angioedema is also challenging due to a lack of clear-cut, reliable diagnostic markers. However, research aims to bridge this gap in understanding. For instance, high levels of cleaved high-molecular-weight kininogen (HK) in plasma may be sensitive for detecting type I HAE (49), and threshold-stimulated kallikrein activity assays may allow for differentiation of histamine-mediated and bradykinin-mediated angioedemas (50). This exploration is crucial, not just as an academic exercise, but as a vital step towards developing effective treatment and management strategies for these conditions (51, 52).
